# Supplementary material for: Identifying and removing the cell-cycle effect from single-cell RNA-Sequencing data
Source: Sci Rep. 2016 Sep 27;6:33892. doi: 10.1038/srep33892 (PMC5037372; doi:10.1038/srep33892)

Supplementary Information for "Identifying and removing the cell-cycle effect from single-cell RNA-Sequencing data"

Martin Barron[1], Jun Li[1,*]

[1]Department of Applied and Computational Mathematics and Statistics, University of Notre Dame, Notre Dame, IN 46556, USA

[*]To whom correspondence should be addressed (email: jun.li@nd.edu).

Supplementary Section S1 - Real dataset 4: mESC Data

This is another of the datasets presented by Buettner et al. to validate scLVM[1]. Different from the three datasets we discuss in the main text, this dataset contains cells from the same cell type, and the cell-cycle stage of each cell is known a priori. The data was generated from mouse embryonic stem cells (mESCs) using the Fluidigm C1 protocol, and the cells were staged for cell-cycle phase (G1, S, G2M) based on sorting of the Hoechst 33342-stained cell area of a flow cytometry (FACS) distribution. It contains gene expression measurements for 9,570 genes and 182 cells, 59 at the G1 stage, 58 at the S stage and 65 at G2M stage. The pre-processed (log-transformed normalized count) data is available in the supplementary material of Buettner et al[1]. The scLVM corrected data is available from the same source. When ccRemover is applied to the data it identifies the first principal component as a cell-cycle effect on the first iteration. Once this effect is removed from the data, no other features are deemed to be cell-cycle related.

To examine ccRemover's effectiveness at removing the cell-cycle effect from the data we use two metrics proposed by Buettner et al.[1]: (1) the existence of distinct clusters related to the cell-cycle stage when the data is projected onto its first two principal components, and (2) the number of significant gene-gene correlations present in the data.

The first metric is based on the same idea as we used in the main text: cells should not cluster according to the cell-cycle stage if the cell-cycle effect has been removed. From the plots of the original data onto its first two principal components there is a clear separation of cells according to the cell-cycle stage represented by the color of each of the points (Supplementary Fig. S1 a). After either ccRemover or scLVM is applied to the data this clear distinction is not observed (Supplementary Fig. S1 b & c). This indicates that the cell-cycle effect has been removed by both scLVM and ccRemover.

The second metric is based on the idea that the cell cycle can lead to many gene-gene correlations in expression (often called gene co-expression) just because the expression of both genes is related to the cell cycle, and thus once the cell-cycle effect is removed there should be a smaller number of significant gene-gene correlations. As such the number of significant gene-gene correlations can be used as a measure of the strength of the cell-cycle effect in the data[1]. We examined the number of gene pairs that have significantly correlated (FDR adjusted, 0.01 level) expression for the original, scLVM corrected and ccRemover corrected data (result shown in Supplementary Table S1). The number of significant gene-gene correlations detected on the scLVM or ccRemover corrected data is significantly lower than that on the original data. Furthermore, as in this data the cell-cycle stages are known a priori, Buettner et al. proposed to calculate the "gold standard gene-gene correlations" by controlling for the cell-cycle stage

and testing for the significance of the coefficient of one gene in a linear model where the expression of the other gene across cells is the response variable. We use a similar approach by fitting the following linear model.

$$y_g = \beta y_{g'} + \mu_{G1}\mathbf{1}_{State=G1} + \mu_{G2M}\mathbf{1}_{State=G2M} + \mu_S\mathbf{1}_{State=S} + \epsilon$$

The number of gold standard significant gene-gene correlations detected was 22,874 (FDR adjusted, 0.01 level), and these correlations are deemed to be "true positives". Gene-gene correlations which are found to be significant on the original dataset, scLVM corrected data, or ccRemover corrected data but not among these gold standard correlations are deemed to be "false positives". The numbers of false positives on different data are shown in Supplementary Table S1. We see that both scLVM and ccRemover dramatically reduce the number of false positives, and that ccRemover leads to less false positives than scLVM (2,284 vs 2,602).

Supplementary Section S2 – Simulation of sparse cell-cycle effects on control genes

ccRemover uses control genes to capture the effects in the data prior to comparing them to the cell-cycle genes to determine which effects are cell-cycle effects. If the cell-cycle effect only influences few control genes, it may not be captured by the principal components and hence missed for detection by ccRemover. We want to investigate how robust ccRemover is to the "sparsity" of the cell-cycle effect on control genes.

We simulate data where we vary the percentage of control genes that are affected by the cell cycle. This is done using a modified version of our simulation model from the main text, where we generated the cell-cycle effect on the control genes Z_{is_j} by $Z_{i1}, Z_{i2}, Z_{i3} \sim N(0, 0.6^2)$. Now, we generate the cell-cycle effect by $Z_{is_j} = Z'_{is_j} * I_i$, where $Z'_{i1}, Z'_{i2}, Z'_{i3} \sim N(0, 0.6^2)$ and $I_i \sim Bernoulli(p)$. That is, only p proportion of control genes are influenced by the cell-cycle effect. The smaller p is, the sparser the cell-cycle effect on control genes is, and the harder the effect is to capture (and hence remove) using ccRemover. Our original simulation in the main text corresponds to $p = 1$. We now decrease the value of p gradually to generate data with sparse cell-cycle effect, and try to find the value of p under which ccRemover stops working on the generated data.

Supplementary Figure S2 shows the performance of ccRemover on data generated with different values of p. We plot the original data (left column) and ccRemover corrected data (right column) on their

first two principal components. Different rows are data with different p values: 50%, 25%, 10%, 8%, and 5%, from top to bottom. We find that when $p \geq 8\%$, the cells (denoted by points) are separated only by the cell type (denoted by the shape), not by the cell-cycle stage (denoted by the color), in the ccRemover corrected data, showing that ccRemover successfully removes the cell-cycle effect. When $p < 8\%$, the cells are separated by both the cell type and the cell-cycle stage, showing that ccRemover does not or does not completely remove the cell-cycle effect. In our simulation, the cell-cycle effect can be described by two principal components, ccRemover is able to capture one or none of them when $p < 8\%$.

ccRemover requires at least 8% of the control genes being influenced by the cell-cycle effect to remove all the cell-cycle effect, in our simulation. This requirement is likely to be satisfied for most real datasets. For example, as we have mentioned in the main text, Buettner et al. found that 44% of the control genes (a set of 6,500 genes not previously associated with the cell cycle) showed significant correlation with at least one cell-cycle gene in the T helper cell data[1].

Interestingly, this percentage, 8%, can be even lower when we have a greater number of genes or a greater number of cells, both of which will help the principal components to capture the cell-cycle effect. To demonstrate this we consider two additional simulations. For the first of these we simulate 10,000 genes and 50 cells, instead of 2,000 genes and 50 cells in our original simulation, and for the second we simulate 2,000 genes and 200 cells. In both cases, ccRemover is able to effectively identify and remove the cell-cycle effect when the proportion of genes affected by the cell-cycle is 4% or greater (Supplementary Fig. S3 and S4).

As a final remark, in all our simulations, the cell-type effect was never incorrectly removed by ccRemover regardless of the proportion of control genes which were affected by the cell cycle. This indicates that it is still safe, although not efficient, to use ccRemover on datasets where the cell-cycle effect is too sparse on the control genes to be detected.

Supplementary Section S3 – Simulation Data with Incomplete and/or Inaccurate Annotations

ccRemover relies on a known set of cell-cycle genes, which are often retrieved from annotation databases. In reality, the annotation databases are always incomplete and inaccurate. In order to examine the performance of ccRemover with incomplete and/or inaccurate annotations we propose additional simulations.

We simulate data according to two additional scenarios. As in the original simulation in the main text, under both scenarios, we simulate data for 50 cells and 2,000 genes, and of these 2,000 genes, 400 are annotated as cell-cycle genes and the others are declared as control genes. In the first scenario, the 400 annotated cell-cycle genes are composed of 200 true cell-cycle genes and 200 true control genes, and all the 1,600 annotated control genes are true control genes. In the second scenario, the 400 annotated cell-cycle genes are composed of 200 true cell-cycle genes and 200 true control genes, and the 1,600 annotated control genes are composed of 200 true cell-cycle genes and 1,400 true control genes. The way we simulate the expression of true control genes and true cell-cycle genes is the same as that in the main text.

ccRemover was applied to data simulated under the two scenarios, and the corrected datasets along with the original dataset were projected onto their first two principal components. The results are displayed in Supplementary Fig. S5 and S6. Under both simulation scenarios, the inaccurate annotations lead to barely any change in the performance of ccRemover. This indicates that ccRemover is quite tolerant to incomplete and/or inaccurate annotations.

References

1. Buettner, F. *et al.* Computational analysis of cell-to-cell heterogeneity in single-cell RNA-sequencing data reveals hidden subpopulations of cells. *Nat. Biotechnol.* **33,** 155–160 (2015).

Supplementary Tables

Supplementary Table S1. The number of significant gene-gene correlations in the mESC data.

**Data**	**Number of Significant Gene-Gene Correlations**	**Number of False Positives**
Original	1,028,686	1,007,111
ScLVM	16,128	2,602
ccRemover	15,358	2,284

Supplementary Figures

Supplementary Figure S1. The data projected onto its first two principal components, with the colors representing the cell-cycle stage of each cell, G1 (red), G2M (blue) and S (green) **(a)** Original data: There are clear distinct clusters related to the cell-cycle stage for the cells. **(b)** scLVM corrected data: The distinct separation of cells by cell-stage is no longer present. **(c)** ccRemover corrected data: Similar to scLVM the cells are no longer separated by the cell-cycle stage.

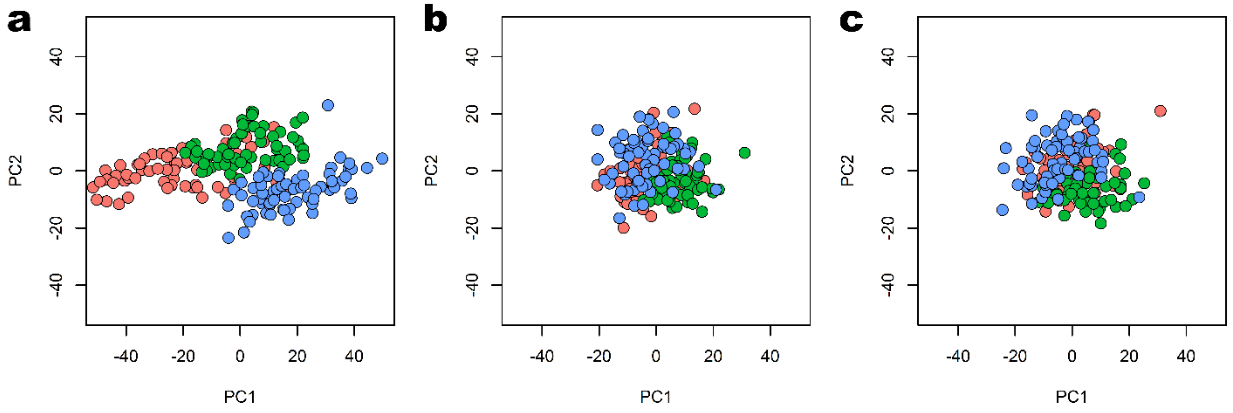

Supplementary Figure S2. Simulated data with 2,000 genes and 50 cells. The rows correspond to differing proportions of the control genes affected by the cell cycle**,** 50%, 25%, 10%, 8% and 5%. The columns represent the different datasets. **(a)** Original data. The cells always split into six clusters corresponding to cell-type and cell-cycle stage combinations. **(b)** ccRemover corrected data. The data splits into two groups corresponding to the cell type until the proportion of control genes affected by the cell cycle falls below 8% for the last row, where separation by the cell-cycle stage is visible.

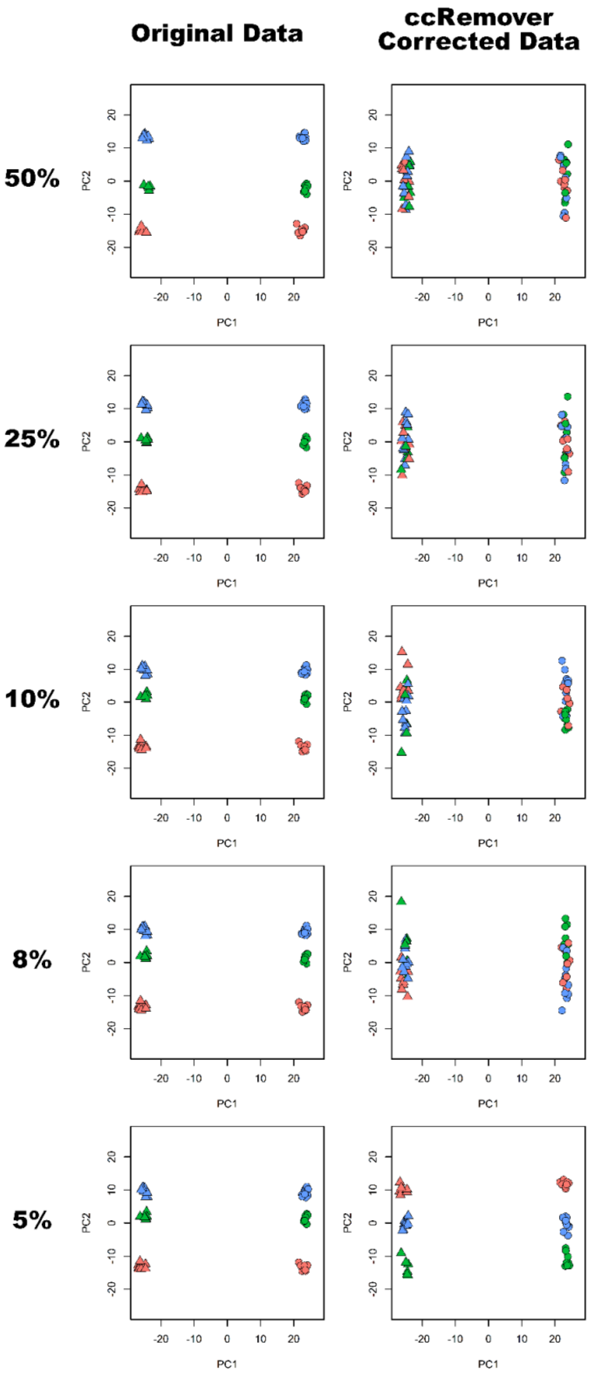

Supplementary Figure S3. Simulated data with 10,000 genes and 50 cells. The rows correspond to differing proportions of the control genes affected by the cell cycle, 25%, 10%, 5%, 4% and 2%. The columns from represent the different datasets. **(a)** Original data. The cells always split into six clusters corresponding to cell-type and cell-cycle stage combinations. **(b)** ccRemover corrected data. The data splits into two groups corresponding to the cell type until the proportion of control genes affected by the cell cycle falls below 4% for the last row, where separation by the cell-cycle stage is visible.

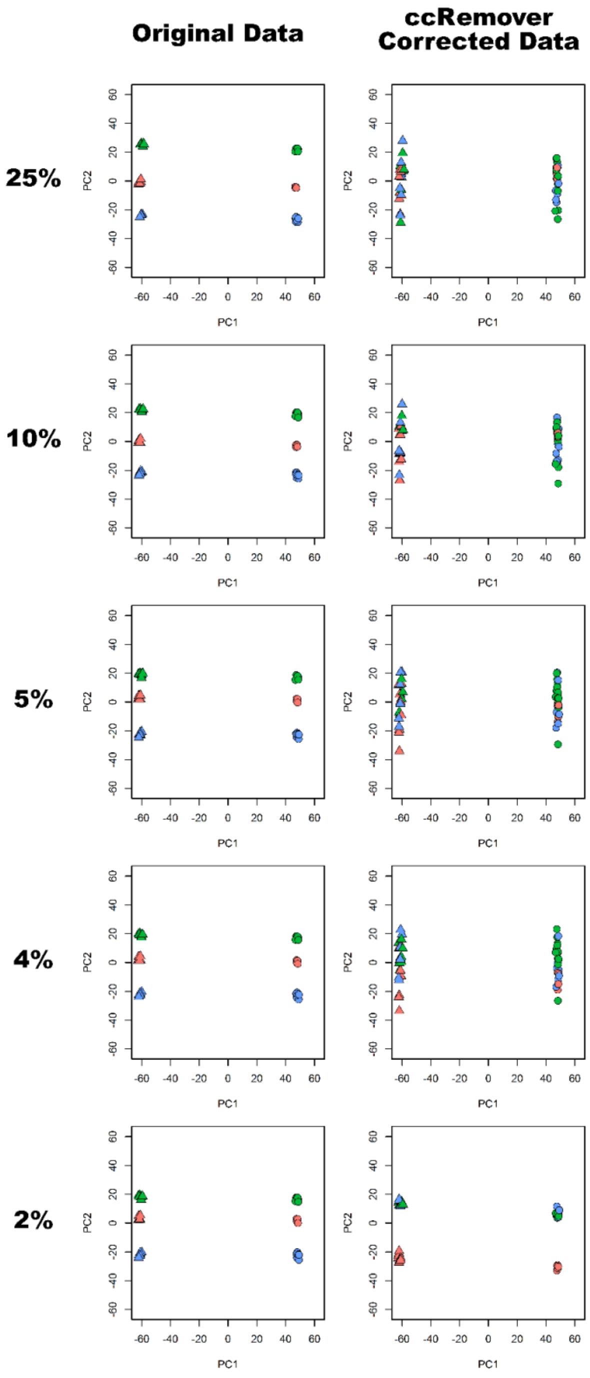

Supplementary Figure S4. Simulated data with 2,000 genes and 200 cells. The rows correspond to differing proportions of the control genes affected by the cell cycle**,** 25%, 10%, 5%, 4% and 2%. The columns represent the different datasets. **(a)** Original data. The cells always split into six clusters corresponding to cell-type and cell-cycle stage combinations. **(b)** ccRemover corrected data. The data splits into two groups corresponding to the cell type until the proportion of control genes affected by the cell cycle falls below 4% for the last row, where separation by cell-cycle stage is visible.

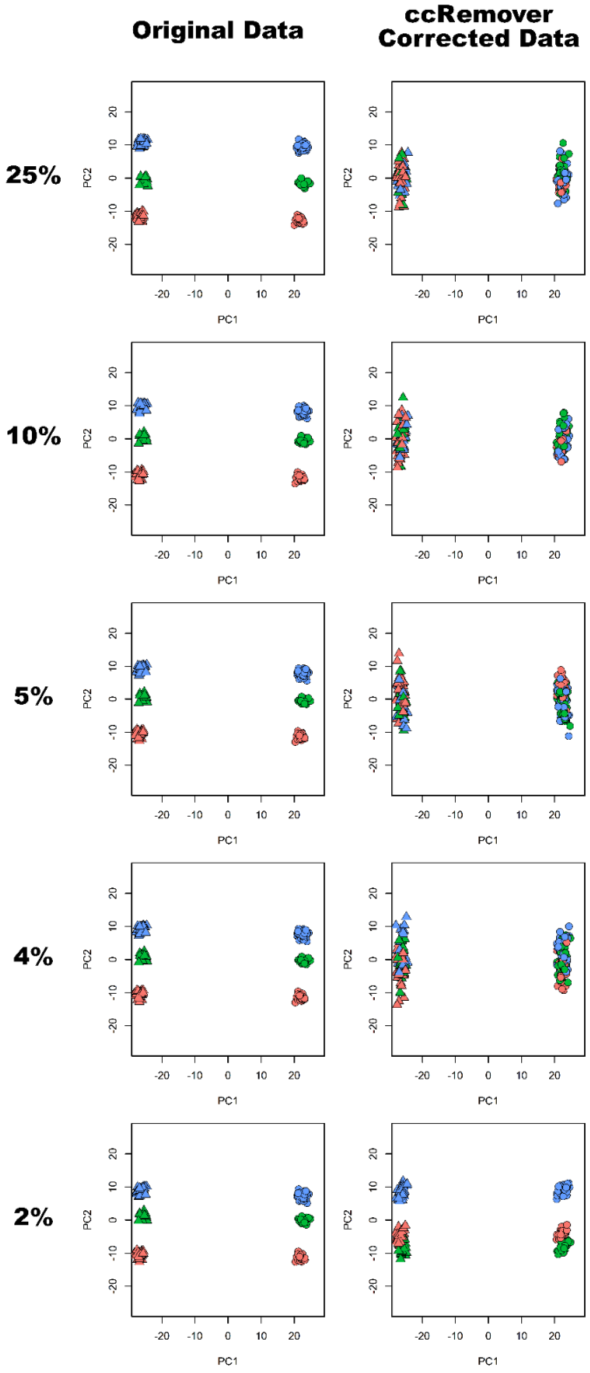

Supplementary Figure S5. Simulated data with 200 cell-cycle genes annotated to the cell-cycle and 200 control genes annotated to the cell cycle. 1,600 control genes as control genes. **(a)** Original data. The data is clustered into six groups corresponding to the combinations of cell type and cell-cycle status. **(b)** ccRemover corrected data. The data splits into two groups corresponding to the cell types.

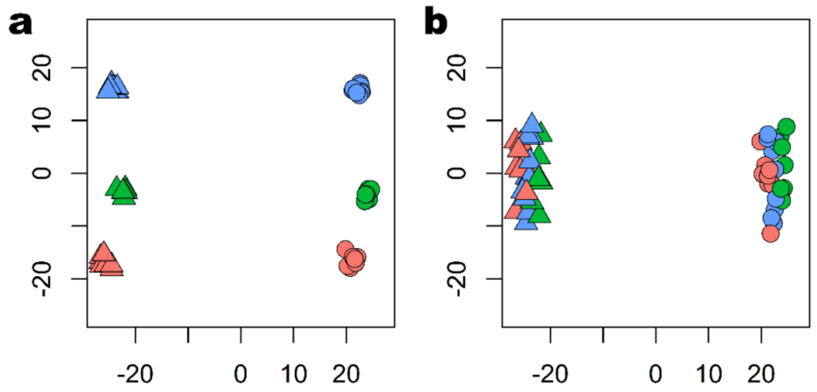

Supplementary Figure S6. Simulated data with 200 cell-cycle genes annotated to the cell cycle and 200 control genes annotated to the cell cycle. 1,400 control genes and 200 cell-cycle genes as control genes. **(a)** Original data. Here the data is clustered into six groups corresponding to the combinations of cell type and cell-cycle status. **(b)** ccRemover corrected data. The data splits into two groups corresponding to the cell types.

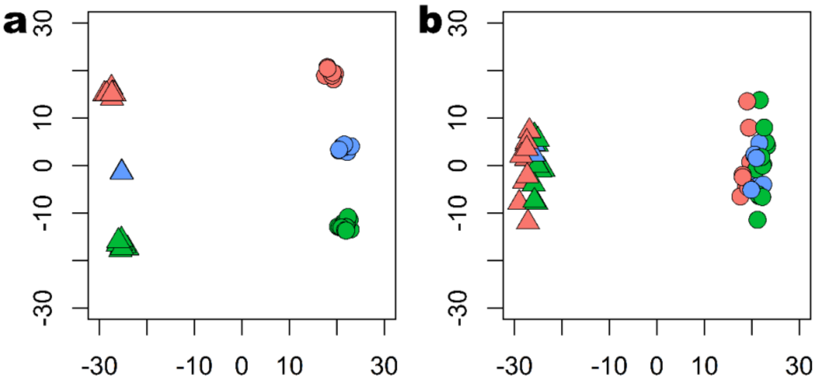

Supplement: Supplementary Information [file srep33892-s1.pdf]
